# Mismatch of populations between randomised controlled trials of perioperative interventions in major abdominal surgery and current clinical practice

**DOI:** 10.1186/s13741-023-00344-w

**Published:** 2023-11-16

**Authors:** Elliott Ridgeon, Rory Shadwell, Alice Wilkinson, Peter M. Odor

**Affiliations:** 1 Department of Anaesthetics and Perioperative Medicine, Wexham Park Hospital, Slough, UK; 2 Department of Anaesthetics and Perioperative Medicine, University College London Hospitals, London, UK; 3 Perioperative Medicine MSc, University College London, London, UK; 4 Department of Critical Care, University College London Hospitals, London, UK; 5 Department of Anaesthetics, University College London Hospitals, London, UK

**Keywords:** Perioperative medicine, Major abdominal surgery, Demographics, Risk, Applicability, Generalisability

## Abstract

**Background:**

Demographics of patients undergoing major abdominal surgery are changing. External validity of relevant RCTs may be limited by participants not resembling patients encountered in clinical practice. We aimed to characterise differences in age, weight, BMI, and ASA grade between participants in perioperative trials in major abdominal surgery and patients in a reference real-world clinical practice sample. The secondary aim was to investigate whether time since trial publication was associated with increasing mismatch between these groups.

**Methods:**

MEDLINE and Embase were searched for multicentre RCTs from inception to September 2022. Studies of perioperative interventions in adults were included. Studies that limited enrolment based on age, weight, BMI, or ASA status were excluded. We compared trial cohort age, weight, BMI, and ASA distribution to those of patients undergoing major abdominal surgery at our tertiary referral hospital during September 2021 to September 2022. We used a local, single-institution reference sample to reflect the reality of clinical practice (i.e. patients treated by a clinician in their own hospital, rather than averaged nationally). Mismatch was defined using comparison of summary characteristics and ad hoc criteria based on differences relevant to predicted mortality risk after surgery.

**Results:**

One-hundred and six trials (44,499 participants) were compared to a reference cohort of 2792 clinical practice patients. Trials were published a median (IQR [range]) 13.4 (5–20 [0–35]) years ago. A total of 94.3% of trials were mismatched on at least one characteristic (age, weight, BMI, ASA). Recruitment of ASA 3 + participants in trials increased over time, and recruitment of ASA 1 participants decreased over time (Spearman’s Rho 0.58 and − 0.44, respectively).

**Conclusions:**

Patients encountered in our current local clinical practice are significantly different from those in our defined set of perioperative RCTs. Older trials recruit more low-risk than high-risk participants—trials may thus ‘expire’ over time. These trials may not be generalisable to current patients undergoing major abdominal surgery, and meta-analyses or guidelines incorporating these trials may therefore be similarly non-applicable. Comparison to local, rather than national cohorts, is important for meaningful on-the-ground evidence-based decision-making.

**Supplementary Information:**

The online version contains supplementary material available at 10.1186/s13741-023-00344-w.

## Background

Demographic characteristics of patients undergoing major surgery are particularly important since they have value for predicting post-operative morbidity. Prognostic factors known to influence patient morbidity and mortality following major surgery include age (Fowler et al. 2019), body weight or body mass index (BMI) (Ri et al. 2018), and the American Society of Anesthesiologists (ASA) score, taken as a summary measure of patient comorbidity status (Hackett et al. 2015). These characteristics are commonly recorded in descriptions of participant samples in randomised controlled trials (RCTs) and are an important indicator of which populations the trial best represents.

For RCTs to be useful to the clinician making evidence-based treatment decisions, trial participants should be representative of real-world patients receiving an intervention in clinical practice. This concept is termed external validity or generalisability. Maximal generalisability occurs when the trial inclusion/exclusion criteria result in participant samples that are highly representative of the real-world patient population (Fogel 2018).

The representativeness of participant characteristics in RCTs of perioperative interventions for major abdominal surgery patients has not been tested. A number of reports have previously highlighted changing demographics of the general population at a national level (National Population Projections – n.d), in terms of patients undergoing all surgery (Alleway et al. 2011) and specifically emergency abdominal surgery (NELA Project Team 2023). The recently published NAP7 (National Audit Project 7) activity survey clearly demonstrates that surgical patients are changing: between NAP5 (2013) and NAP7 (2021), patients are on average older, higher BMI, and more likely to be ASA 3 +  (Kane et al. 2023). However, trial samples are fixed from the time of participant recruitment,those within the trial cannot be changed, even though later samples of patients receiving that same treatment outside the trial may be increasingly different over months and years. It is therefore possible that current treatment decisions, guidelines, and care pathways are based on evidence generated through examining historic populations that effectively no longer exist. Indeed, multiple highly cited perioperative meta-analyses include RCTs over 20 years old (Deng et al. 2020), (Probst et al. 2017), (Frauenknecht et al. 2019).

Comparisons of RCT populations to reference cohorts in terms of demographics or risk have traditionally used national or international patient databases. One review of trials in selected perioperative domains used national cohorts matched chronologically to RCTs (i.e. a trial from 2005 was compared to database patients from 2005) (Lindsay et al. 2020). However, the database patient is not, in fact, the ‘real-world’ patient. The clinician makes evidence-based treatment decisions for patients in their own hospital, not for patients pooled from hospitals across the country. Nor do they treat patients from history—the degree of matching of a 2005 trial to a patient hospitalised in 2005 reflects only the generalisability of the trial at that time, not in the present day.

The primary aim of this review is therefore to demonstrate a method for evaluating closeness of demographic matching of RCT participants, and hence generalisability, to patients encountered in a clinician’s current real-world practice in an example institution. The review will focus on perioperative interventions in major abdominal surgery. The secondary aim is to investigate whether the passage of time is associated with increasing mismatch between RCT participant attributes and those of unselected patients.

## Methods

This systematic review was conducted and is reported in concordance with the Preferred Reporting Items for Systematic Reviews and Meta- Analyses (PRISMA) guidelines (Page et al. 2020). Local ethical and research governance approvals were granted for access to anonymised patient data for purposes of extracting comparator sample data. The protocol for this study did not meet the criteria for registration on the International Prospective Register of Systematic Reviews (PROSPERO) because of the open inclusion approach to trials with respect to outcome measures and controls.

### Search strategy

Searches were conducted in MEDLINE and Embase via OvidSP, from inception to September 2022. Search strategies were adapted from those used in previous systematic reviews addressing related topics (Deng et al. 2020), (Boet et al. 2021), to include major abdominal surgery and perioperative care. A combination of relevant keywords and medical subject heading terms were used (full search terms and strategy are detailed in Appendix 1). No limits were placed on language of publication.

### Study inclusion and exclusion criteria

Studies were included if they were multicentre randomised controlled trials investigating interventions in the pre-, intra-, or post-operative period for adult patients (≥ 18 years) undergoing major abdominal surgery, either elective or emergency.

Major abdominal surgery was defined as any surgical procedure involving the abdomen, classified as major, x-major, or complex, including laparoscopic, open, and robotic approaches. Severity coding was based on the reference manual for the AXA Specialist Procedure Codes, used to grade the magnitude of surgical procedures in UK independent hospitals (available at https://specialistforms.onlineapps.axahealth.co.uk) and for pre-operative risk stratification (Protopapa et al. 2014). Colorectal surgery, hepatobiliary surgery, vascular surgery, gynaecology, and urology procedures were included. Multicentre studies are defined as those involving more than one hospital.

Controls and outcomes were not limited. Trials were included regardless of their control group (usual care, placebo, alternative intervention) and their outcome measures (mortality, morbidity, biomarker changes, etc.). The primary outcome measure was descriptive statistics for the composition of each population sample in trials.

We excluded studies investigating interventions in patients having non-abdominal surgery or obstetric surgery; studies with a defined age limit other than adult (i.e. other than ≥ 18 years); restrictions on inclusion for BMI, weight, or ASA; reanalysis/follow-up of previous RCT results; studies without cohort demographic information given; those investigating a solely surgical intervention (e.g. comparison of different surgical techniques); those investigating solely chemotherapy or radiotherapy interventions; those conducted solely in the setting of transplant surgery; pilot/feasibility studies; and those conducted only in a single hospital.

### Choice of demographic measures

We chose age, weight, BMI, and ASA score as the focus for our study based on their known link to perioperative risk (1–3) and their common reporting in RCTs (versus, e.g. ethnicity, which is poorly reported in trials (Lindsay et al. 2020), and other various summary risk measures, e.g. Charlson Comorbidity Index)), acknowledging that these are not the only determinants of patient risk/outcome.

### Comparator (local) cohort

The electronic health record system was searched to identify all patients undergoing major abdominal surgery at University College London Hospitals (a tertiary referral hospital in London) over one year. A single 12-month period from September 2021 to September 2022 was used as the comparator sample, chosen to reflect the most accurate, recent, and therefore representative sample of patients at the host institution. Major abdominal surgery was defined using the same terms as for the systematic review. Records were anonymised, and data were collected on patient age, body weight, BMI, ASA score, and surgical specialty. We used a local, single-institution sample rather than a national database specifically to reflect the real-world practice of clinicians who work locally, not nationally.

### Study selection

Search results were combined into online systematic review software (Ouzzani et al. 2016), and duplicates were removed. Title and abstract screening was performed by three authors. Each article required a minimum of two reviewer decisions before proceeding to the next stage. Differences in opinion were resolved by consensus discussion. Full texts of remaining studies were assessed for eligibility.

### Data extraction

Data from trials were extracted using a pre-designed data capture spreadsheet. We collected the following information from eligible trials, from both intervention and control groups: age of patients, body weight of patients, BMI of patients, ASA score distribution, primary trial focus (ileus, thromboprophylaxis, fluid therapy, analgesia, transfusion/iron therapy, nutrition, antibiotics), trial result (significant for either benefit or harm/non-significant or neutral), surgical specialty (colorectal, hepatobiliary, upper gastrointestinal, gynaecology, urology, vascular, mixed), recruitment at UK hospitals (non-UK, UK inclusive, solely UK), trial sample size (number of patients and number of centres), and years since publication.

Trials were divided into subgroups by their primary focus, result, surgical subspecialty, size (divided at the median trial size for all included studies), and years since publication (divided at the median number of years for all included studies).

### Quality assessment

Trial quality was assessed using the NIH (US) study quality assessment tool for controlled intervention studies (https://www.nhlbi.nih.gov/health-topics/study-quality-assessment-tools). This tool was chosen to allow pragmatic and rapid overview of trials, since the methodology of the review as a whole does not specifically require assessment of quality and bias in the traditional way.

### Data synthesis and statistical analysis

We reported continuous variables as mean and standard deviation (SD) or median, interquartile range (IQR), and range. We reported categorical variables as counts and percentages.

We compared trial cohort demographics to those of our local cohort to detect differences in population age, body weight, BMI, and ASA score distribution. Trials were compared to patients undergoing procedures of the same surgical specialty (i.e. trials in colorectal surgery were compared to patients undergoing colorectal surgery). Trials enrolling mixed specialties were compared to the total real-world patient sample. Differences between means for continuous variables were assessed using a two-sided Welch’s *T*-test for samples of unequal variance. Where trials reported summary statistics other than mean (SD), we converted results from median and range and/or interquartile range to mean and standard deviation using standardised methods (Higgins et al. 2022), (Wan et al. 2014), (Hozo et al. 2005). Differences between proportions of ordinal or binary variables were assessed using the chi-squared test.

We determined the number and percentage of trials in each subgroup with cohort demographics matching those of the local comparator sample. Differences between subgroups in terms of numbers matching the comparator sample were assessed using the chi-squared test.

We also determined the number and percentage of trials with demographics substantially different from the comparator sample (rather than simply significantly different to a statistical degree). Substantial differences were defined as mean age + / − 10 years versus comparator, mean weight + / − 10 kg versus comparator, and mean BMI in different band from comparator (i.e. if the comparator cohort mean BMI was 28 (overweight), a trial BMI of < 25 (normal), or > 30 (obese) would be deemed substantially different).

To account for variable reporting of demographics between trials, we also determined the number of mismatches as a fraction of demographics reported (i.e. for a trial reporting patient age, BMI, and ASA, a statistical match in age but mismatch elsewhere would give a result of 1/3). We pooled these results when assessing trial subgroups.

We assessed the ability of years since trial publication to predict any cohort mismatch (i.e. significant difference between trial and local cohort in any of the following: age, body weight, BMI, ASA score distribution), or substantial cohort mismatch using receiver-operating characteristic (ROC) curve analysis. Correlation of publication year with individual parameters was assessed using Spearman’s Rho, after trial parameters were pooled by year using inverse variance weighting.

Trials are expected to be heterogeneous by virtue of the design of the research question—no restriction by intervention or outcome has been applied. On this basis, no formal meta-analysis was undertaken. Statistical analysis was performed using the Real Statistics Resource Pack software (Release 7.6) (Copyright 2013–2021, Charles Zaointz, www.real-statistics.com).

Our study is summarised as follows, using the PICO framework:

- Patients: Adults undergoing major abdominal surgery
- Intervention group: RCT populations (participants in our defined set of trials of perioperative interventions in major abdominal surgery)
- Comparator group: Local population (adults undergoing major abdominal surgery at our centre, September 2021 to September 2022)
- Outcomes: The presence of statistical mismatch between intervention and comparator groups based on age, weight, BMI, or ASA distribution

## Results

### Description of included studies

**Fig. 1:**
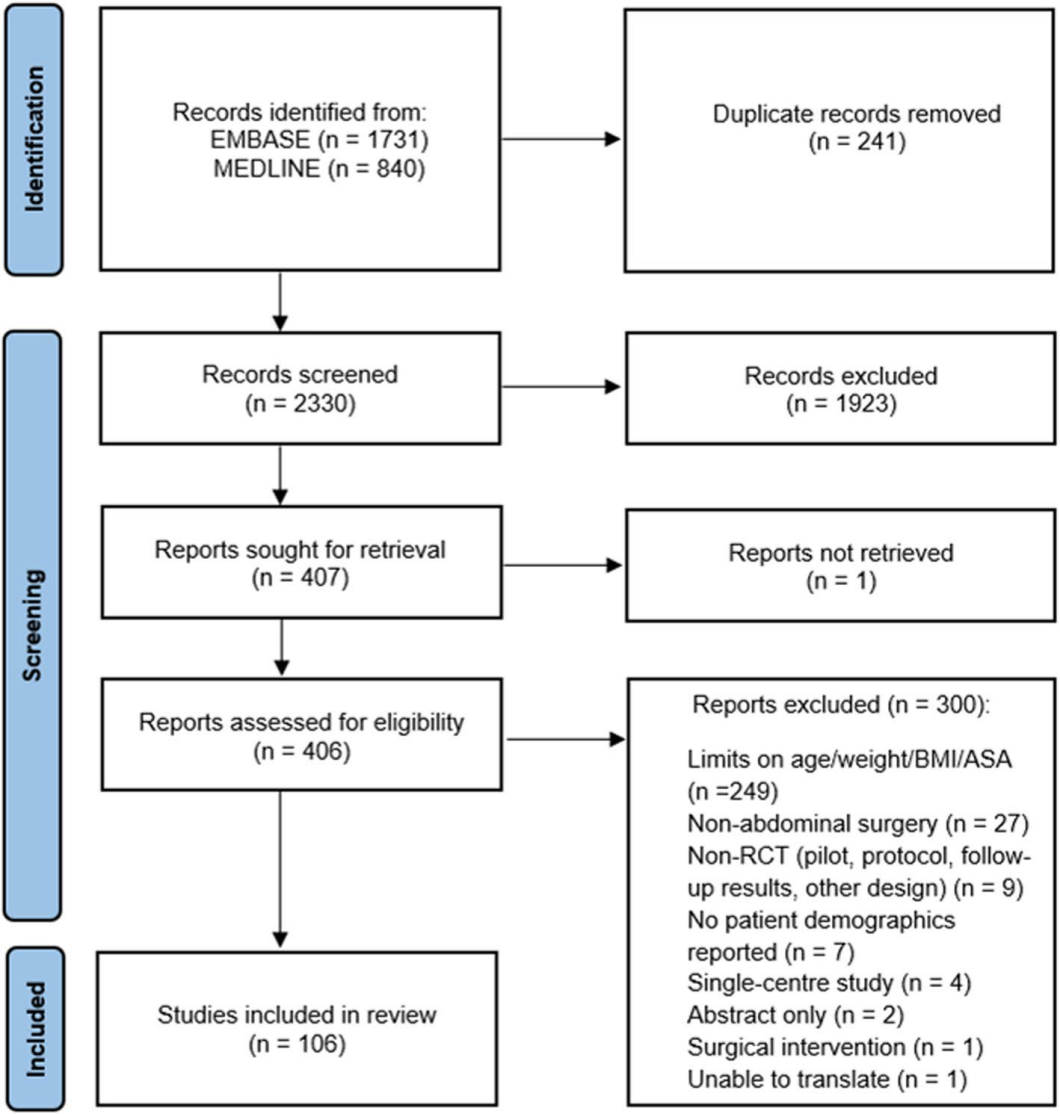
PRISMA flow diagram for study selection process

Our literature search retrieved 2330 unique citations. Of these, 116 RCTs, including a total of 44,499 trial participants, fulfilled the inclusion criteria and were included in the analysis. Median (IQR [range]) number of participants included in each RCT was 253 (121–489 [30–4352]), with number of recruiting hospitals of 6 (3–13 [2-61]). Results of search and selection processes are shown in Fig. 1.

Trials were published a median (IQR [range]) of 13.4 (5–20 [0–35]) years ago. The newest trial was published in September 2022 and the oldest in October 1987.

The most common investigative subjects were antibiotics in 20 RCTs (18.9% of all RCTs), nutrition in 12 trials (11.3%), and post-operative ileus in 10 (9.4%). Most trials were undertaken in participants undergoing colorectal (46, 43.4%) or gynaecological (17, 16.0%) surgery, with a further 23 (21.7%) trials in mixed surgical specialties. Fifty-four (50.9%) trials reported a significant result for their primary outcome. Only 6 (5.7%) studies were conducted exclusively in the UK, with a further 3 (2.8%) including UK centres. The remaining majority recruited entirely outside the UK. Risk of bias was assessed as low in 26 (24.5%) studies, fair in 19 (17.9%), and high in 61 (57.5%). Characteristics of all included studies are shown in Table 1.

**Table 1:** Characteristics of all included trials

									***Age (years)***	***Wgt (kg)***	***BMI***	***ASA***	***Statistical mismatches***				***Substantial mismatches***		
	**Years since publication**	**Topic of trial**	**Surgical specialty**	**Outcome**	**Centres**	**UK centres**	**Risk of bias**	**Trial size**	**Mean (SD)**	**Mean (SD)**	**Mean (SD)**	**% 3 + **	**Age**	**Wgt**	**BMI**	**ASA**	**Age**	**Wgt**	**BMI **
*Auer 2022 *Auer et al. 2022)	0.0	Thrombo-prophylaxis	Colorectal	NS	12	No	High	614	61.1 (12.9)	-	28.4 (5.6)	-	Yes↑	-	Yes↑	-	Yes	-	No
*Fafaj 2022* Fafaj et al. 2022)	0.3	Catheter	Colorectal	NS	6	No	High	491	60 (12.6)	-	26.1 (3.7)	28.1	Yes↑	-	No	Yes↓	No	-	No
*Gao 2022* Gao et al. 2022)	0.5	Nutrition	Colorectal	S	11	No	Fair	229	60.1 (11.3)	62.4 (7.8)	22.9 (3.1)	-	Yes↑	Yes↓	Yes↓	-	No	Yes	Yes
*Aoyama 2022* Aoyama et al. 2022)	0.6	Nutrition	UGI	NS	8	No	High	123	63.7 (12.2)	46.7 (6.7)	-	-	No	Yes↓	-	-	No	Yes	-
*Hakovirta 2022* Hakovirta et al. 2022)	0.6	Inflammatory mediators	Vascular	NS	9	No	High	38	73.5 (9.0)	-	-	-	Yes↑	-	-	-	Yes	-	-
*Cao 2021* Cao et al. 2021)	0.8	Somatostatin	HPB	S	6	No	Fair	199	58.5 (11.0)	-	23 (3)	-	Yes↑	-	Yes↓	-	No	-	Yes
*de Waal 2021* Waal et al. 2021)	1.0	Goal-directed therapy	Mixed	NS	2 +	No	High	482	65.4 (11.4)	78.2 (15.6)	25.9 (4.4)	38.6	Yes↑	No	Yes↓	Yes↑	Yes	No	No
*Marschalek 2021* Marschalek et al. 2531)	1.2	Hormone therapy	Gynae	NS	2	No	Fair	103	62.7 (10.0)	-	27.1 (4.4)	-	Yes↑	-	No	-	Yes	-	No
*Papp 2021* Papp et al. 2021)	1.6	Antibiotics	Colorectal	S	4	No	High	529	66.3 (12.2)	-	27.2 (4.4)	-	Yes↑	-	No	-	Yes	-	No
*Hortu 2020* Hortu et al. 2020)	2.2	Local anaesthetic	Gynae	S	2	No	Low	108	52.2 (6.2)	-	29.7 (4.6)	-	Yes↑	-	No	-	No	-	No
*Mulder 2020* Mulder et al. 2020)	2.3	Antibiotics	Colorectal	S	6	No	Fair	78	67.2 (8.5)	-	26.8 (4.9)	28.2	Yes↑	-	No	No	Yes	-	No
*Boggett 2020* Boggett et al. 2020)	2.4	Neuromuscular blockade	Mixed	NS	4	No	High	350	54.5 (15.9)	-	29.2 (6.1)	24.4	No	-	Yes↑	Yes↓	No	-	No
*Espin Basany 2020* Espin Basany et al. 2020)	2.4	Antibiotics	Colorectal	S	5	No	High	536	70.8 (12.6)	-	27.6 (4.1)	48.9	Yes↑	-	Yes↑	-	Yes	-	No
*Bohlin 2020* Bohlin et al. 2020)	2.6	Smoking cessation	Gynae	S	2 +	No	High	651	48 (10.0)	-	-	1.8	No	-	-	Yes↓	No	-	-
*Futier 2020* Futier et al. 2020)	2.7	IV fluids	Mixed	NS	20	No	Low	775	68.5 (7.0)	81.5 (12.8)	27.5 (5.5)	48.6	Yes↑	Yes↓	No↑	Yes↑	Yes	No	No
*Bretagnol 2020* Bretagnol et al. 2010)	2.9	Bowel prep	Colorectal	S	8	No	Fair	178	63.5 (3.1)	-	25 (0.8)	-	Yes↑	-	No	-	Yes	-	No
*Linecker 2020* Linecker et al. 2015)	3.0	Omega 3	HPB	NS	3	No	Low	261	58.2 (15.8)	-	24.8 (3.7)	-	Yes↑	-	Yes↓	-	No	-	Yes
*De Milliano 2019* Milliano et al. 2020)	3.1	Hormone therapy	Gynae	S	9	No	High	54	39.8 (5.9)	-	26.2 (5.5)	-	Yes↓	-	No	-	No	-	No
*Bruintjes 2019* Bruintjes et al. 2019)	3.5	Neuromuscular blockade	Urology	NS	2	No	Fair	96	56.2 (9.9)	-	26.6 (2.9)	-	Yes↓	-	No	-	No	-	No
*Habib 2019* Habib et al. 2019)	3.7	Antiemetics	Mixed	S	23	No	Low	702	46.3 (10.9)	-	-	-	Yes↓	-	-	-	No	-	-
*Karanicolas 2018* Karanicolas et al. 2018)	4.2	Wound infusion catheters	HPB	S	2	No	Low	153	62.7 (12.6)	-	-	-	Yes↑	-	-	-	Yes	-	-
*Springer 2018* Springer et al. 2018)	4.3	Post-op ileus	Colorectal	NS	2	No	Low	118	65 (14)	-	28.6 (6)	94.9	Yes↑	-	Yes↑	Yes↑	Yes	-	No
*Kranke 2018* Kranke et al. 2018)	4.3	Antiemetics	Mixed	S	29	No	Low	1147	48.5 (14)	-	30.3 (8.6)	-	Yes↓	-	Yes↑	-	No	-	Yes
*Sang 2018* Sang et al. 2018)	4.3	Thrombo-prophylaxis	Gynae	S	5	No	High	625	53.7 (10.3)	-	24.9 (3.8)	-	Yes↑	-	Yes↓	-	No	-	No
*de Leede 2018* Leede et al. 2018)	4.6	Post-op ileus	Mixed	NS	12	No	Low	1941	65.8 (12.9)	-	25.6 (6.8)	17.9	Yes↑	-	Yes↓	Yes↓	Yes	-	No
*Peters 2018* Peters et al. 2018)	4.6	Nutrition	Colorectal	NS	6	No	Low	265	68.5 (8.9)	-	26.2 (4.1)	15.5	Yes↑	-	No	Yes↓	Yes	-	No
*Boden 2018* Boden et al. 2018)	4.7	Pre-hab	Mixed	S	3	No	Low	432	64.3 (14.7)	-	28.4 (6)	36.4	Yes↑	-	Yes↑	Yes↑	No	-	No
*Chanques 2017* Chanques et al. 2017)	5.0	Sedation	Mixed	S	3	No	Low	137	67.8 (14.1)	-	25.8 (4.8)	-	Yes↑	-	Yes↓	-	Yes	-	No
*Brunschot 2017* Özdemir-van Brunschot et al. 2018)	5.3	Neuromuscular blockade	Urology	S	2	No	Low	34	-	-	25.5 (3.7)	-	-	-	Yes↓	-	-	-	No
*Keeler 2017* Keeler et al. 2017)	5.7	Iron	Colorectal	NS	7	UK only	Fair	116	73.9 (9.0)	75.7 (12.2)	-	37.1	Yes↑	No	-	No	Yes	No	-
*Burden 2017* Burden et al. 2017)	5.7	Nutrition	Colorectal	S	6	UK only	Low	101	69.8 (11.6)	-	25.7 (4.7)	23.1	Yes↑	-	No	Yes↓	Yes	-	No
*Loozen 2017* Loozen et al. 2017)	5.8	Antibiotics	HPB	NS	6	No	Low	150	53.2 (10.4)	-	-	-	No	-	-	-	No	-	-
*Topsoee 2016* Topsoee et al. 2016)	6.2	TXA	Gynae	S	4	No	Low	332	48.5 (8.6)	-	25.4 (4.7)	0.3	No	-	Yes↓	Yes↓	No	-	No
*Piljic 2016* Piljic et al. 2016)	6.3	IV fluids	Vascular	S	2	No	High	60	69 (8.0)	-	-	-	Yes↑	-	-	-	Yes	-	-
*Jaber 2016* Jaber et al. 2016)	6.5	NIV	Mixed	S	20	No	Low	293	63.4 (13.8)	-	27.2 (10.1)	-	Yes↑	-	No	-	No	-	No
*Atkinson 2016* Atkinson et al. 2016)	6.6	Post-op ileus	Colorectal	NS	5	UK only	Fair	402	66.2 (12.9)	-	27.5 (5.1)	22.1	Yes↑	-	Yes↑	Yes↓	Yes	-	No
*Fushida 2015* Fushida et al. 2015)	7.0	Tiotropium	UGI	NS	15	No	High	82	-	-	22.5 (2.6)	-	-	-	Yes↓	-	-	-	Yes
*Hamza 2015* Hamza et al. 2015)	7.7	Nutrition	HPB	S	3	UK only	High	37	65.2 (9.9)	-	24.4 (5.6)	-	Yes↑	-	Yes↓	-	Yes	-	Yes
*van den Heijkant 2015* Heijkant et al. 2015)	7.8	Post-op ileus	Colorectal	S	2	No	High	120	66.5 (10.1)	78.5 (11.9)	26.5 (4.5)	10.8	Yes↑	Yes↓	No	Yes↓	Yes	No	No
*Pestana 2014* Pestana et al. 2014)	8.1	Goal-directed therapy	Mixed	NS	6	No	Low	142	72.3 (11.6)	72.9 (14.8)	26.8 (4.4)	51.4	Yes↑	Yes↓	No	Yes↑	Yes	No	No
*Regimbeau et al. (2014)* Regimbeau et al. 2014	8.2	Antibiotics	HPB	NS	17	No	High	414	55.5 (12.4)	-	-	-	Yes↑	-	-	-	No	-	-
*Kakkar 2014* Kakkar et al. 2014)	8.3	Thrombo-prophylaxis	Mixed	NS	2 +	-	High	4352	61.9 (11.9)	-	25.6 (8.5)	-	Yes↑	-	Yes↓	-	No	-	No
*Vedovati 2014* Vedovati et al. 2014)	8.5	Thrombo-prophylaxis	Colorectal	S	5	No	Fair	225	64.8 (9.0)	-	25.3 (3.4)	-	Yes↑	-	No	-	Yes	-	No
*Young 2013* Young et al. 2013)	9.0	Communication	Colorectal	NS	23	No	Fair	756	67.8 (12.2)	-	-	-	Yes↑	-	-	-	Yes	-	-
*Giger-Pabst 2013* Giger-Pabst et al. 2013)	9.4	Nutrition	Colorectal	NS	6	No	High	108	64.1 (12.7)	75.4 (10.2)	-	-	Yes↑	No	-	-	Yes	No	-
*Muller 2012* Muller et al. 2012)	10.0	Post-op ileus	Colorectal	S	3	No	Fair	79	60.5 (13.5)	-	-	29.1	Yes↑	-	-	Yes↑	No	-	-
*Stott 2012* Stott et al. 2012)	10.1	Laxatives	Colorectal	S	2	No	Fair	45	65.6 (14.3)	-	-	-	Yes↑	-	-	-	Yes	-	-
*Phoolcharoen 2012* Phoolcharoen et al. 2012)	10.3	Antibiotics	Gynae	NS	2	No	Low	320	45.4 (7.0)	-	-	-	Yes↓	-	-	-	No	-	-
*Barlow 2011* Barlow et al. 2011)	11.6	Nutrition	UGI	S	3	UK only	High	121	64 (11.1)	73 (13.3)	25.4 (4)	46.3	No	No	Yes↓	Yes↓	No	No	No
*Wattchow 2009* Wattchow et al. 2009)	12.9	Post-op ileus	Colorectal	NS	3	No	Low	210	62.1 (14)	77 (18.7)	-	-	Yes↑	No	-	-	Yes	No	-
*Meyhoff 2009* Meyhoff et al. 2009)	13.0	Oxygen therapy	Mixed	NS	14	No	Low	1395	64 (20.4)	71.5 (13.4)	25 (4)	19.1	Yes↑	Yes↓	Yes↓	Yes↓	No	No	No
*Shimizu 2010* Shimizu et al. 2010)	13.1	Antibiotics	Colorectal	NS	4	No	High	91	69.7 (8.5)	-	-	8.8	Yes↑	-	-	Yes↓	Yes	-	-
*Trivedi 2007* Trivedi et al. 2007)	13.2	Hormone therapy	Gynae	S	5	No	High	98	30.9 (9.7)	55.1 (10)	-	-	Yes↓	Yes↓	-	-	Yes	Yes	-
*Muller 2009* Muller et al. 2009)	13.6	Enhanced recovery	Colorectal	S	4	No	Fair	151	60.6 (9.6)	-	25.1 (2.8)	27.8	Yes↑	-	No	Yes↓	No	-	No
*Ludwig 2008* Ludwig et al. 2008)	13.9	Post-op ileus	Colorectal	S	55	No	Fair	654	59.8 (14)	-	28.4 (6.3)	-	Yes↑	-	Yes↑	-	No	-	No
*Godet 2008* Godet et al. 2008)	14.4	IV fluids	Vascular	NS	7	No	High	60	73 (7.8)	73.6 (8.6)	-	-	Yes↑	Yes↓	-	-	Yes	No	-
*Del Rio 2008* Rio et al. 2008)	14.5	Antibiotics	HPB	NS	2 +	No	High	209	53.6 (14.6)	-	-	-	No	-	-	-	No	-	-
*Lassen 2008* Lassen et al. 2008)	14.7	Nutrition	UGI	NS	5	No	High	447	64 (13.9)	-	-	-	No↑	-	-	-	No	-	-
*Contant 2007* Contant et al. 2007)	14.8	Bowel prep	Colorectal	NS	13	No	High	1354	67 (12.5)	-	-	12.2	Yes↑	-	-	Yes↓	Yes	-	-
*Aqua 2007* Aqua et al. 2007)	15.0	Analgesia	Gynae	S	21	No	Low	331	42.7 (9.3)	-	-	-	Yes↓	-	-	-	No	-	-
*Skanberg 2007* Skanberg et al. 2007)	15.5	Blood transfusion	Colorectal	NS	7	No	High	642	71.7 (9.5)	-	-	-	Yes↑	-	-	-	Yes	-	-
*Han-Geurts 2007* Han-Geurts et al. 2007)	15.6	Post-op ileus	Colorectal	NS	3	No	High	128	63.6 (14.9)	-	27 (7.9)	-	Yes↑	-	No	-	Yes	-	No
*Ellison 2007* Ellison et al. 2007)	15.7	Communication	Urology	NS	3	No	High	270	54 (9.8)	-	-	-	Yes↓	-	-	-	No	-	-
*Chermesh 2007* Chermesh et al. 2007)	15.7	Probiotics	Colorectal	NS	4	No	High	30	35.6 (11.9)	64.5 (14.3)	-	-	Yes↓	Yes↓	-	-	Yes	No	-
*Wichmann 2007* Wichmann et al. 2007)	15.7	Nutrition	Mixed	S	4	No	High	256	59.3 (11.5)	72.6 (13.4)	25.2 (3.9)	-	Yes↑	Yes↓	Yes↓	-	No	No	No
*Rasmussen 2006* Rasmussen et al. 2006)	16.2	Thrombo-prophylaxis	Mixed	S	5	No	High	343	66.3 (11.4)	71.5 (14)	-	-	Yes↑	Yes↓	-	-	Yes	No	-
*White 2006* White et al. 2006)	16.7	Antiemetics	Mixed	NS	2	No	High	205	38.5 (13.5)	102 (35)	37.5 (10.5)	-	Yes↓	Yes↓	Yes↑	-	Yes	Yes	Yes
*Valverde 2006* Valverde et al. 2006)	16.7	Bowel prep	Colorectal	NS	20	No	High	467	66 (12.6)	-	-	-	Yes↑	-	-	-	Yes	-	-
*Ming-Tsan 2005* Lin et al. 2005)	17.1	Nutrition	UGI	S	2	No	High	48	67.1 (8.6)	57.2 (9.6)	-	-	Yes↑	Yes↓	-	-	No	Yes	-
*Viscusi 2006* Viscusi et al. 2006)	17.2	Post-op ileus	Mixed	S	37	No	High	444	56.5 (11.9)	-	28.5 (6.7)	-	No↑	-	Yes↑	-	No	-	No
*Fa-Si-Oen 2005* Fa-Si-Oen et al. 2005)	17.2	Bowel prep	Colorectal	NS	5	No	High	250	68.4 (10.0)	-	-	-	Yes↑	-	-	-	Yes	-	-
*Bucher 2005* Bucher et al. 2005)	17.9	Bowel prep	Colorectal	S	2	No	Fair	153	63 (9.9)	-	-	11.8	Yes↑	-	-	Yes↓	Yes	-	-
*Malan 2005* Malan et al. 2005)	18.0	Analgesia	Gynae	S	2 +	-	High	264	44.6 -	81.2 (22.7)	-	-	-	Yes↓	-	-	-	No	-
*Wolff 2004* Wolff et al. 2004)	18.0	Post-op ileus	Colorectal	S	34	No	High	469	60.5 (11.4)	-	28.2 (5)	-	Yes↑	-	Yes↑	-	No	-	No
*van Hilten 2004* Hilten et al. 2004)	18.4	Blood transfusion	Mixed	NS	19	No	Low	1051	66.5 (11.3)	-	-	-	Yes↑	-	-	-	Yes	-	-
*Nordin 2004* Nordin et al. 2004)	18.6	Anaesthetic technique	Colorectal	S	3	No	High	138	56.6 (14.6)	-	24.4 (2.8)	-	Yes↑	-	Yes↓	-	No	-	Yes
*Nordin 2003* Nordin et al. 2003)	19.1	Anaesthetic technique	Colorectal	S	10	No	Fair	616	56 (13.7)	-	25 (4)	-	Yes↑	-	No	-	No	-	No
*Buppasiri 2004* Buppasiri et al. 2004)	19.1	Decontamination	Gynae	S	3	No	Low	299	43.9 (7.4)	59.3 (9.6)	24.5 (3.6)	-	Yes↓	Yes↓	Yes↓	-	No	Yes	Yes
*Chongsomchai 2002* Chongsomchai et al. 2002)	20.0	Antibiotics	Gynae	S	2	No	Low	321	43.6 (6.3)	-	23.9 (3.4)	-	Yes↓	-	Yes↓	-	No	-	Yes
*Rigg 2002* Rigg et al. 2002)	20.6	Epidural	Mixed	NS	25	No	Low	915	69 (11.0)	-	-	-	Yes↑	-	-	-	Yes	-	-
*Tonelli 2002* Tonelli et al. 2002)	20.7	Antibiotics	Mixed	NS	2 +	No	High	476	63.6 (13.0)	-	-	-	Yes↑	-	-	-	No	-	-
*McLeod 2001* McLeod et al. 2001)	22.1	Thrombo-prophylaxis	Colorectal	NS	10	No	High	1349	51 (17.5)	-	-	-	No	-	-	-	No	-	-
*Takala 2000* Takala et al. 2000)	22.7	CVS support	Mixed	NS	13	UK incl	High	432	62.5 (13.4)	70 (14)	-	-	Yes↑	Yes↓	-	-	No	No	-
*Zanella 2000* Zanella and Rulli 2000)	22.8	Antibiotics	Colorectal	NS	14	No	High	615	65 (11.2)	-	-	-	Yes↑	-	-	-	Yes	-	-
*Verspyck 2000* Verspyck et al. 2000)	23.3	Hormone therapy	Gynae	S	10	No	High	56	41.4 (1.8)	-	24.1 (0.9)	-	Yes↓	-	Yes↓	-	No	-	Yes
*Jian 1999* Jian et al. 1999)	23.4	Nutrition	Colorectal	S	4	No	High	120	55 (12.6)	59.7 (9.5)	-	-	Yes↑	Yes↓	-	-	No	Yes	-
*Valverde 1999* Valverde et al. 1999)	23.7	Bowel prep	Colorectal	S	18	No	High	523	68 (12.5)	-	-	-	Yes↑	-	-	-	Yes	-	-
*Cutillo 1999* Cutillo et al. 1999)	23.8	Nutrition	Gynae	S	3	No	High	122	53.3 (14.1)	-	-	-	Yes↑	-	-	-	No	-	-
*Vercellini 1998* Vercellini et al. 1998)	24.2	Hormone therapy	Gynae	S	4	No	Fair	123	45.9 (3.5)	-	23.2 (3.1)	-	Yes↓	-	Yes↓	-	No	-	Yes
*Milsom 1998* Milsom et al. 1998)	24.7	Antibiotics	Colorectal	NS	61	No	High	317	59.5 (15.1)	78 (17)	-	-	Yes↑	No	-	-	No	No	-
*Heiss 1997* Heiss et al. 1997)	25.2	Blood transfusion	Colorectal	S	3	No	High	70	58.8 (10.0)	-	-	-	Yes↑	-	-	Yes↑	No	-	-
*O'Hara 1997* O’Hara et al. 1997)	25.4	Analgesia	Gynae	S	3	No	High	191	43.4 (10.7)	76.6 (22.7)	-	-	Yes↓	No	-	-	No	No	-
*Johnson 1997* Johnson et al. 1997)	25.7	Analgesia	Gynae	S	3	No	High	190	41 (11.8)	75.3 (18.4)	-	-	Yes↓	No	-	-	No	No	-
*Vickers 1995* Vickers and Paravicini 1995)	27.4	Analgesia	Mixed	NS	26	UK incl	Fair	523	52 (15)	70.5 (12.5)	-	-	Yes↓	Yes↓	-	-	No	No	-
*Stankov 1995* Stankov et al. 1995)	27.8	Analgesia	UGI	S	5	No	Low	100	48.8 (14.5)	75.5 (13.7)	-	-	Yes↓	No	-	-	Yes	No	-
*Taylor 1994* Taylor et al. 1994)	27.9	Antibiotics	Colorectal	S	13	No	High	327	66.6 (12.9)	-	-	-	Yes↑	-	-	-	Yes	-	-
*Stewart 1995* Stewart et al. 1995)	28.7	Antibiotics	Colorectal	S	13	UK incl	High	326	66.6 (12.8)	-	-	-	Yes↑	-	-	-	Yes	-	-
*Houbiers 1994* Houbiers et al. 1994)	28.7	Blood transfusion	Colorectal	NS	16	No	High	697	68.5 (14.3)	-	-	-	Yes↑	-	-	-	Yes	-	-
*Friess 1994* Fiess et al. 1994)	28.8	Octreotide	HPB	S	18	No	High	246	51.2 (9.7)	-	-	-	No	-	-	-	No	-	-
*Gipponi 1993* Gipponi et al. 1993)	29.8	IVIG/sepsis	Colorectal	S	3	No	High	159	65.5 (8.5)	-	-	-	Yes↑	-	-	-	Yes	-	-
*Andaker 1992* Andaker et al. 1992)	30.6	Antibiotics	Colorectal	NS	9	No	High	517	66.5 (14)	68.5 (11.5)	-	-	Yes↑	Yes↓	-	-	Yes	No	-
*Arnaud 1992* Arnaud et al. 1992)	30.7	Antibiotics	Colorectal	NS	19	No	Fair	208	66 (12.0)	68 (13)	-	-	Yes↑	Yes↓	-	-	Yes	No	-
*Rotman 1991* Rotman et al. 1991)	30.9	Antibiotics	Mixed	NS	24	No	High	1254	50.9 (18.7)	-	-	-	Yes↓	-	-	-	No	-	-
*DiPiro 1989* DiPiro et al. 1989)	33.8	Antibiotics	Colorectal	NS	3	No	High	195	53.9 (17.5)	-	-	-	No	-	-	-	No	-	-
*Rotman 1989* Rotman et al. 1989)	34.4	Antibiotics	Mixed	NS	2 +	-	High	3137	47.6 (17.8)	-	-	-	Yes↓	-	-	-	No	-	-
*Walker 1988* Walker et al. 1988)	34.9	Antibiotics	Colorectal	NS	7	UK only	High	213	63.5 (10.6)	-	-	-	Yes↑	-	-	-	Yes	-	-

*S* significant. *NS* non-significant. *Wgt* weight (kg)

↑Indicates a mismatch where a trial parameter is higher than reference cohort

↓Indicates a mismatch where a trial parameter is lower than reference cohort. Parameters for which no data were available are denoted by -

**Fig. 2:**
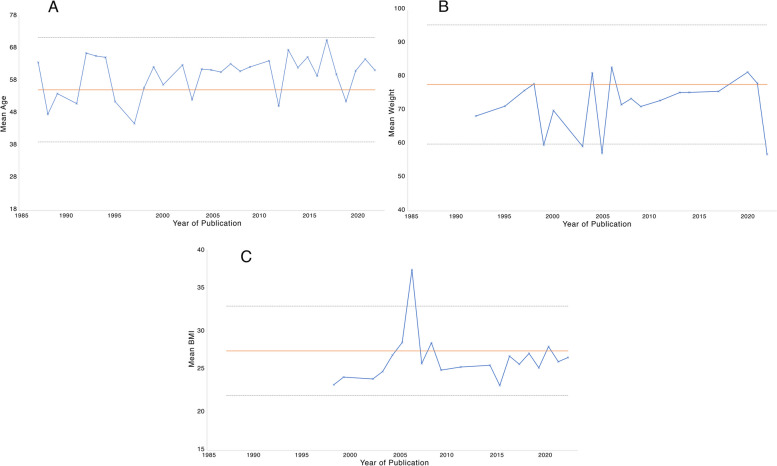
Changes in trial demographics over time. Solid orange lines represent mean of comparator sample for given parameter, dotted lines represent mean + / − 1 standard deviation. **A** Mean age of trial participants over time. **B** Mean weight of trial participants over time. **C** Mean BMI of trial participants over time

**Fig. 3:**
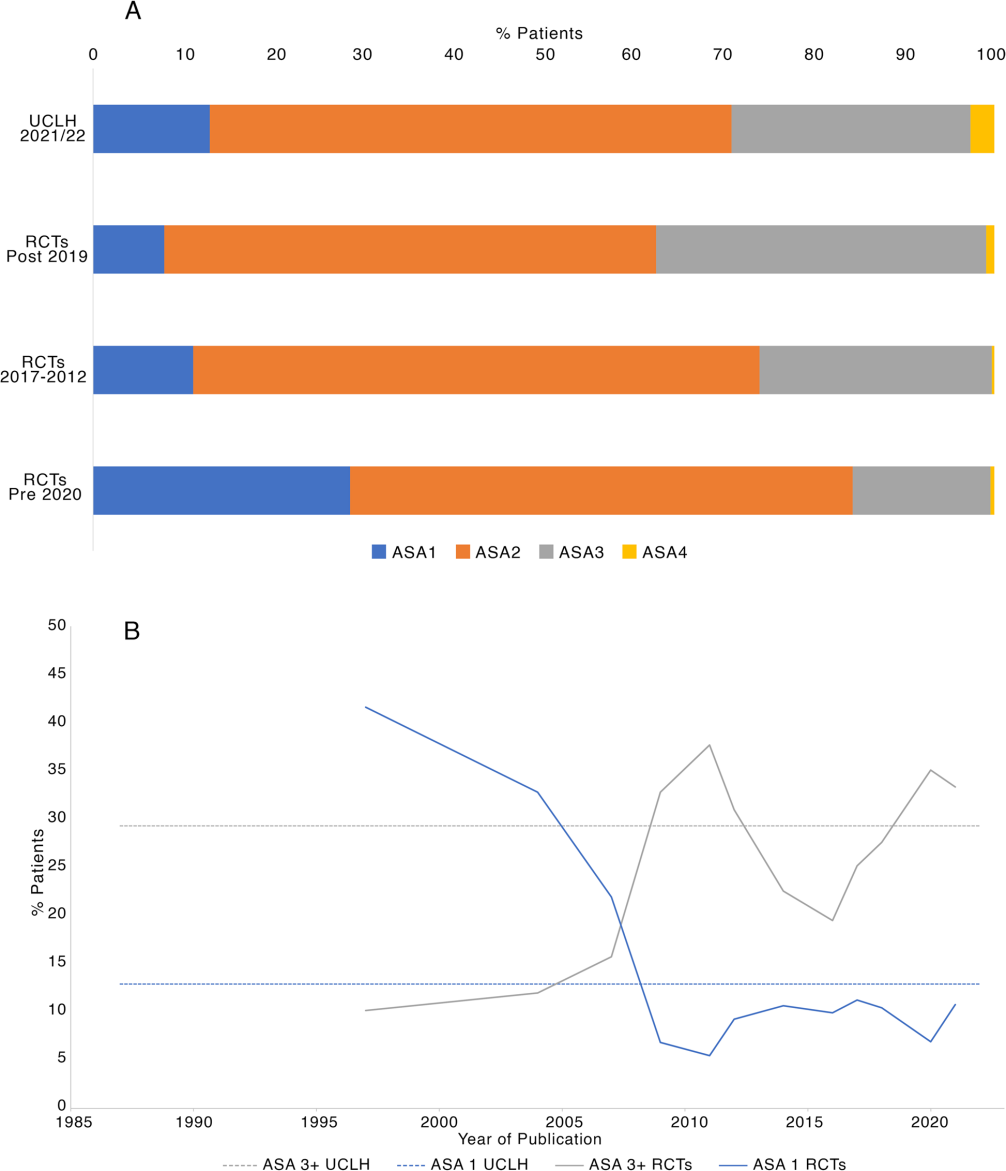
**A** Changing distribution of ASA scores of participants in RCTs over time, with real-world reference cohort top. **B** Change in percentage of ASA 1 and ASA 3 + participants recruited over time. Trendlines are 2-period moving average. Solid grey, % of participants ASA 3 + recruited to perioperative RCTs. Dotted grey, % of ASA 3 + patients in UCLH reference cohort. Solid blue, % of participants ASA 1 recruited to perioperative RCTs. Dotted blue, % of ASA 1 patients in UCLH reference cohort

Participant age was reported by 103 (97.2%) trials, weight by 29 (27.4%), BMI by 49 (46.2%), and ASA by 25 (23.5%). Changes in mean trial participant age, weight, and BMI over time are shown in Fig. 2. Changes in ASA distribution over time are shown in Fig. 3.

### Comparator (local) cohort

**Fig. 4:**
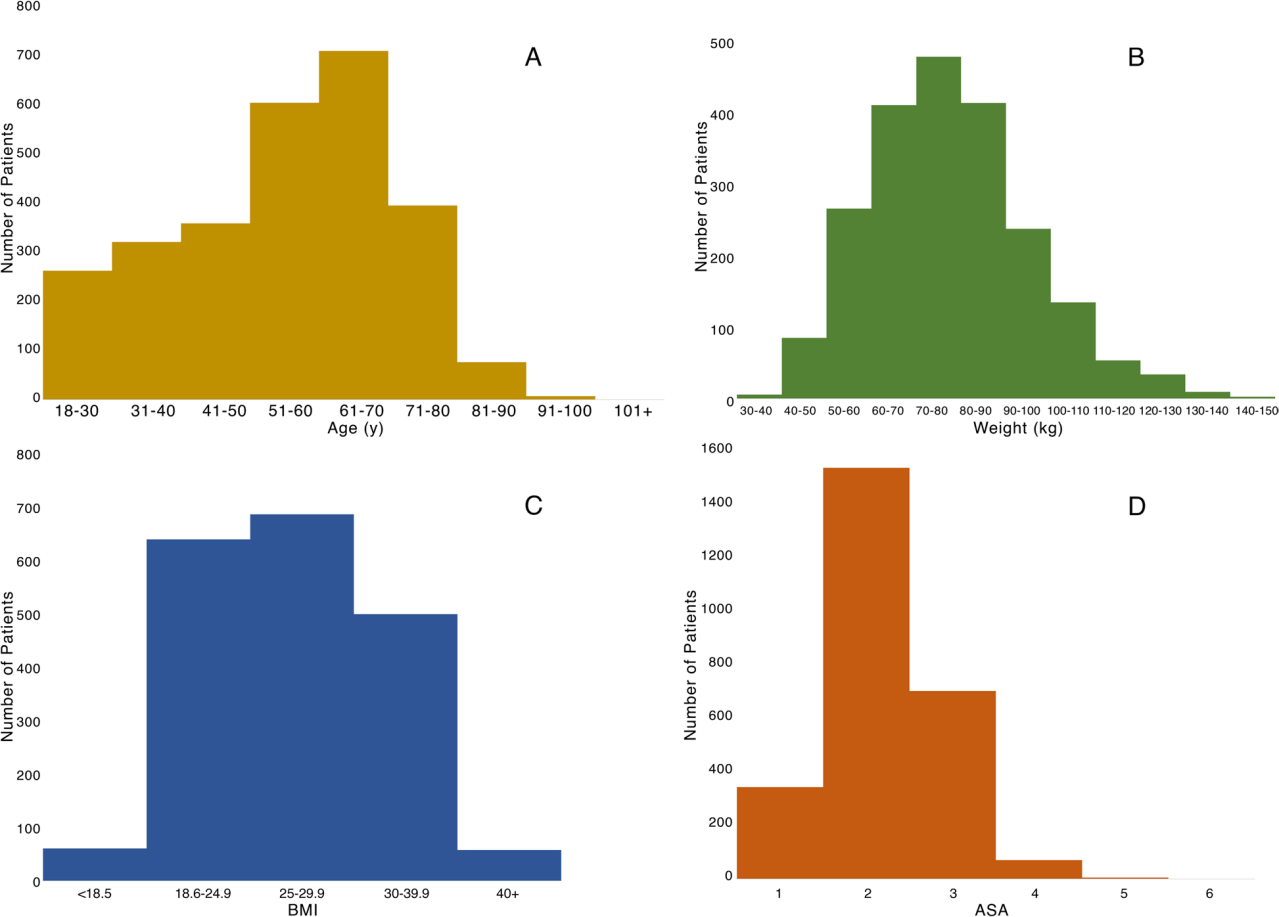
Distribution of demographic parameters for real-world patient cohort. **A** Age. **B** Weight. **C** BMI. **D** ASA score

A total of 2792 patients underwent major abdominal surgery at University College London Hospitals (UCLH) in the year September 2021 to September 2022. Most patients had urological (774, 27.7%), gynaecological (725, 26.0%), or colorectal (613, 22.0%) procedures. Mean (SD) age for all included patients was 55.1 (16.0) years, mean (SD) weight was 77.8 (17.8) kg, and mean (SD) BMI was 27.4 (5.6). Of patients with recorded ASA score, 773 (29.3%) were ASA 3 or higher. Age, weight, BMI, and ASA distributions of this cohort are shown in Fig. 4.

### Mismatch between trial sample and local patient characteristics

One-hundred and six trials reported on a total of 205 demographic parameters. One-hundred trials (94.3%) had at least one demographic characteristic that was significantly different from the local patient cohort. Mean age of patients was mismatched in 91 studies (88.3% of those reporting age), weight in 20 (69.0% of those reporting weight), BMI in 31 (63.3% of those reporting BMI), and ASA distribution in 22 (91.7% of those reporting ASA). Substantial mismatches in age, weight, and BMI were seen in 50 (48.5%), 7 (24.1%), and 12 (24.5%) trials respectively.

Patients were younger, of lower weight, and of lower BMI in 23 (22.3% of trials reporting age), 29 (100% of trials reporting weight), and 30 (61.2% of trials reporting BMI) trials respectively. Sixteen (64.0%) trials reporting ASA had a lower percentage of ASA 3 + patients than the local reference cohort.

Trials older than the median 13.4 years since publication were more likely to report mismatches, with 73 (84.9%) parameters in this subgroup significantly different from the local population versus 91 (76.5%) in trials published more recently, *p* = 0.001. This relationship reversed for substantial mismatches (41.9% pre-median vs 46.2% post-median, *p* = 0.002). Older trials were significantly more likely to report a lower BMI than that found in the reference cohort (6 (46.2%) trials vs 14 (38.9%) trials, *p* < 0.001). All four trials older than 13.4 years which reported ASA were mismatched in terms of ASA distribution, and three of these recruited a lower percentage of patients with ASA score > 3 than the reference cohort.

However, after inverse variance weighting, minimal correlation was seen between year of publication and increasing age, weight, or BMI (Spearman’s Rho 0.36, 0.20, and 0.24, respectively). Percentage of ASA 3 + patients in a trial showed moderate positive correlation with year of publication, with Spearman’s Rho 0.58. Percentage of ASA 1 patients in a trial showed moderate negative correlation with year of publication, Rho − 0.44.

ROC analysis demonstrated poor prediction of substantial mismatch in any parameter by years since publication of trial, with area under the curve of 0.64.

### Subgroup analysis

When compared to a subset of the local cohort undergoing colorectal surgery, 44 (95.7%) trials in this specialty were statistically mismatched for any one of age, weight, BMI, or ASA distribution. Substantial mismatch in age, weight, and BMI was seen in 23 (50.0%), 3 (30.0%), and 2 (10.5%) trials reporting these demographics respectively.

One-hundred percent of 23 trials in mixed specialty cohorts were statistically mismatched for any one of age, weight, BMI, or ASA distribution. Substantial mismatch in age, weight, or BMI was seen in 13 (56.5%).

Mixed studies had a higher rate of parameters statistically mismatched than colorectal studies (47 (88.8%) vs 70 (78.7%), *p* < 0.001) but a lower rate of substantial parameter mismatches (19 (36.5%) vs 40 (41.0%), *p* < 0.001).

Trials investigating analgesia, blood transfusion or iron therapy, and nutrition had the lowest rates of statistical mismatch for parameters reported (7 (70.0%), 6 (75.0%), and 21 (75.0%), respectively). Trials of analgesia and thromboprophylaxis had the lowest rates of substantial mismatch in parameters (1 (10.0%) and 3 (27.3%), respectively).

Outcome of a trial (significant or non-significant) showed no relationship with rate of statistically mismatched demographic parameters or substantially mismatched demographic parameters (any statistical mismatch 86 (80.4%) vs 78 (79.6%), *p* = 0.35, substantial mismatch 44 (41.1%) vs 47 (48.0%), *p* = 0.08).

Smaller trials (≤ 253 participants) were less likely to show mismatch overall compared to larger trials (78 (74.3%) vs 86 (86%) parameters, *p* < 0.001) but more likely to show substantial mismatch (53 (50.5%) vs 38 (38.0%), *p* = 0.006).

Subgroup results are summarised in Table 2.

**Table 2:** Trial characteristics and mismatches of demographics by subgroup

				***Statistical mismatch***			***Substantial mismatch***		
***Group***	***n* (%)**	**Trial sample size, median (IQR [range])**	**Years since publication, median (IQR [range])**	**Trials, *n* (%)**	**Parameters, *n* (%)**	***p***	**Trials, *n* (%)**	**Parameters, *n* (%)**	***p***
***Surgical specialty***									
*Hepatobiliary*	8 (7.5)	204 (152–250 [37–414])	6.8 (3.9–9.8 [0.8–28.8])	5 (62.5)	8 (72.7)		4 (50)	5 (45.5)	
*Vascular*	3 (2.8)	60 (49–60 [38-60])	6.3 (3.5–10.3 [0.6–14.4])	3 (100)	4 (100)		3 (100)	3 (75)	
*Gynaecology*	17 (16.0)	191 (108–321 [54–651])	15 (4.3–23.3 [1.2–25.7])	17 (100)	25 (78.1)		8 (47.1)	10 (31.3)	
*Colorectal*	46 (43.4)	227 (122–522 [30–1354])	15.1 (6.9–23.3 [0–34.9])	44 (95.7)	70 (78.7)		40 (87)	40 (44.9)	
*Upper gastrointestinal*	6 (5.7)	111 (87–123 [48–447])	13.2 (8.1–16.5 [0.6–27.8])	5 (83.3)	7 (58.3)		5 (83.3)	5 (41.7)	
*Urology*	3 (2.8)	96 (65–183 [34–270])	5.3 (4.4–10.5 [3.5–15.7])	3 (100)	3 (75)		0 (0)	0 (0)	
*Mixed specialty*	23 (21.7)	482 (347–1099 [137–4352])	13 (4.6–19.5 [1.0–34.4])	23 (100)	47 (88.7)		13 (56.5)	19 (35.8)	
***Topic of trial***									
*Post-op ileus*	10 (9.4)	306 (122–463 [79–1941])	11.5 (7–15 [4.3–18.0])	10 (100)	21 (84)		7 (70)	11 (44)	
*Antibiotics*	20 (18.9)	321 (209–476 [78–3137])	24.7 (14.5–30.7[1.6–34.9])	17 (85)	22 (75.9)		11 (55)	12 (41.4)	
*Antiemetics*	3 (2.8)	702 (454–925 [205–1147])	4.3 (4–10.5 [3.7–16.7])	3 (100)	6 (100)		2 (66.7)	4 (66.7)	
*IV fluids/GDT*	5 (4.6)	142 (60–482 [60–775])	6.3 (2.7–8.1 [1.0–14.4])	5 (100)	12 (80)		5 (100)	8 (53.3)	
*Blood transfusion/iron*	5 (4.7)	642 (116–697 [70–1051])	18.4 (15.5–25.2 [5.7–28.7])	5 (100)	6 (75)		5 (100)	5 (62.5)	
*Thromboprophylaxis*	6 (5.7)	620 (411–1168 [225–4352])	8.4 (5.3–14.3 [0–22.1])	5 (83.3)	9 (81.8)		3 (50)	3 (27.2)	
*Bowel prep*	6 (5.7)	359 (196–509 [153–1354])	17 (15.3–17.8 [2.9–23.7])	6 (100)	8 (88.9)		6 (100)	8 (88.9)	
*Nutrition*	12 (11.3)	122 (106–234 [37–447])	10.5 (5.5–16.1 [0.5–23.8])	11 (91.7)	21 (75)		9 (75)	13 (46.4)	
*Analgesia*	6 (5.7)	228 (190–314 [100–523])	25.6 (19.8–27 [15–27.8])	6 (100)	7 (70)		1 (16.7)	1 (10)	
*Other*	33 (31.1)	159 (98–350 [30–1395])	7 (3.1–18.6 [0.3–29.8])	32 (97)	52 (81.3)		24 (72.7)	26 (40.6)	
***Outcome***									
*Significant*	54 (50.9)	190.5 (111–332 [34–1147])	13.4 (4.8–19.7 [0.5–29.8])	53 (98.1)	86 (80.4)	0.35	37 (68.5)	44 (41.1)	0.08
*Non-significant*	52 (49.10	335 (148–622 [30–4352])	13.7 (5.4–20.6 [0–34.9])	47 (90.4)	78 (79.6)		36 (69.2)	47 (48)	
***Trial size***									
< = *253*	53 (50)	-	11.6 (5.3–18.6 [0.5–34.9])	49 (92.5)	78 (74.3)	< 0.001	42 (79.2)	53 (50.5)	0.006
> *253*	53 (50)	-	15.0 (4.6–20.6 [0–34.4])	51 (96.2)	86 (86)		31 (58.5)	38 (38)	
***Years since publication***									
*More recent than 13.4*	53 (50)	529 (376–729 [34–4352])	-	52 (98.1)	91 (76.5)	< 0.001	42 (79.2)	55 (46.2)	0.002
*Older than 13.4*	53 (50)	299 (159–517 [30–3137])	-	48 (90.6)	73 (84.9)		31 (58.5)	36 (41.9)	

*IQR* interquartile range. *GDT* goal-directed therapy

*P*-values are for differences in numbers of mismatched demographic parameters between groups, calculated by chi-squared test

## Discussion

### Key findings

We conducted a systematic review of the literature to identify mRCTs of perioperative interventions in patients undergoing major abdominal surgery, excluding those with limitations to recruitment based on patient age, weight, BMI, or ASA. We compared the cohorts studied in these trials to a reference cohort of patients undergoing surgery of the same specialty and found that 94.3% were statistically mismatched on at least one characteristic (patient age, weight, BMI, ASA). Older trials were significantly more likely to be mismatched from the reference cohort than more recent trials in terms of ASA, and these older trials reported lower percentages of ASA 3 + participants recruited. Recruitment of ASA 3 + participants increased as the year of trial publication approached 2022 (positive correlation), but recruitment of ASA 1 participants decreased (negative correlation), demonstrating a divergence of RCT populations from current patient populations in older trials.

### Relation to previous studies

This is the first study to assess matching of RCT cohorts without recruitment limitations to a local reference cohort across multiple risk-associated demographic parameters. A study in 2020 (Lindsay et al. 2020) used a purposive sampling strategy to identify perioperative trials in seven surgical specialties and assessed reporting of age, sex and ethnicity, and similarity to reference cohorts from national databases covering five specialties (of which only colorectal resection was a major abdominal surgical group). Our work expands on this by adding other risk-linked demographics, focusing on major abdominal surgery beyond colorectal procedures, and utilising a locally derived but large reference cohort. Ours is also the first study to assess cohort matching in these demographics over time, using a current reference cohort of patients (as opposed to a reference cohort drawn from the same period as study recruitment).

A time-trend analysis published in 2019 (Fowler et al. 2019) describes the advancing age of the surgical population when compared to the general population and estimates that one-fifth of over 75 s will undergo surgery annually by 2030. This highlights the importance of our finding that more than 20% of perioperative RCTs in major abdominal surgery recruited patients significantly younger than those in our reference cohort. With increasing surgical age as described, an ever-greater number of trials will become mismatched, and the effective expiry date of a piece of clinical evidence will shorten.

The recently published NAP7 activity survey demonstrates a surgical population that is ageing, increasing in BMI, and more likely to be comorbid (as described by increasing numbers of patients ASA 3 +) when compared to past surveys (Kane et al. 2023). We have shown that perioperative RCTs in major abdominal surgery follow this pattern in terms of ASA, with increasing recruitment of participants ASA 3 + towards the present day and decreasing recruitment of patients ASA 1. However, when looking backwards in time from the present, we obviously see the reverse as the trendlines of Fig. 3B diverge: trials become increasingly demographically distant from current patient populations (both NAP7 and our reference cohort).

While changes in real-world patient demographics may be an explanation for our findings, selection bias may also play an important role. A 2015 review (Kennedy-Martin et al. 2015) of studies assessing selection bias in cardiology, mental health, and oncology by comparing RCT cohorts to real-world cohorts found that 71.2% reported misrepresentation of the general population in their respective specialty. This prior work does not consider time (years since publication) as a factor. Whether caused by demographic drift or changes in trial design over time, our finding of increased mismatch in older trials remains. Cardiology studies on selection bias consistently showed a higher-risk population encountered in everyday clinical practice than in RCTs, which is similar to our findings. In oncology studies on selection bias, two-thirds found that over 50% of real-world patients would be ineligible for RCT participation. We focused on trials with ‘open recruitment’, i.e. without restrictive inclusion/exclusion criteria in terms of age/weight/BMI/ASA, which removes this as a driver of selection bias in our review.

### Strengths and limitations

The strength of this study is the use of a locally derived reference cohort, rather than one drawn from national registries or databases. Perioperative clinicians commonly work in one hospital or one area and treat patients in that same setting. Hence, although a trial population may differ from a national population, the real question on the mind of the clinician is likely to be as follows: ‘how well does this trial match the patients I actually treat in my own hospital?’ Our study shows that the answer to this question is ‘probably poorly’, particularly with respect to ASA status. This question also implicitly requires that the reference cohort of patients compared to trials is a current one, i.e. one made up of patients recently or currently presenting for surgery. A further strength of our study is the matching to a such a patient population (local, current) rather than matching to one from the same period as the trials in question. The clinician in London in the present day is likely to value evidence applicable to their own perioperative patients found in London in the present day; they are unlikely to be interested in applicability to an average theoretical national patient, patients from 10 years ago, or patients in a centre distant and disconnected from their own. However, the clinician elsewhere could substitute their local patient population into our methodology, to answer the question meaningfully for them (which could include the finding of good matching between their local patients and those in RCTs).

While our local reference cohort is similar to that described nationally in NAP7 (Kane et al. 2023) (UCLH mean age 55.1 years and BMI 27.4 vs NAP7 median age 52.8 and BMI 26.7), it is limited to patients undergoing major abdominal surgery, and no national registry for such a group exists for comparison. However, the UK National Emergency Laparotomy Audit (NELA) report for 2020–2021 (NELA Project Team 2023) shows a mean patient age of 63.9 years (estimated from values in Table 2), and the colorectal subgroup of the Perioperative Quality Improvement Project (PQIP) database shows a mean patient age of 64 years (Bedford et al. 2022). These are different from our local cohort and highlight the challenges of generalising national registry findings to local practice. Reviewing the degree of matching of pooled multicentre RCT cohorts to relevant up-to-date local patient, populations is thus important.

A second key strength lies in the systematic approach to identification of trials and the exclusion of those with limits on their recruitment based on age, weight, BMI, or ASA. Real-world populations do not present for surgery with such limits, and so trials (or reviews of trials) excluding older, heavier, more obese, and more unwell patients are likely to be less applicable to current surgical patients. Indeed, a previous study (Lindsay et al. 2020) showed that over a third of perioperative trials excluded or were biased against older people, and 4.5% excluded patients based on ASA status. Our study demonstrates that, even with an ‘open’ recruitment approach, trial participants are likely to be different from local real-world patients.

Our choice of demographic variables is both a strength and a limitation: age, weight, BMI, and ASA status are commonly (although not universally) reported by perioperative RCTs, which allows us to build a large dataset across time. However, these are not the only variables that are associated with morbidity risk in major abdominal surgery and may provide an incomplete description of patients. Additionally, evidence of association of these with risk is mixed: one review focusing on elderly surgical patients found no relationship between increasing age or ASA status and complications (Watt et al. 2018), but another investigating abdominal surgery found a strong effect of age on increasing likelihood of mortality and morbidity (Massarweh et al. 2009). This latter work supports our definition of substantial mismatch as an age difference of + / − 10 years, with an approximately 4% absolute increase in complications for each additional 10 years of patient age—trial populations different from our local population by more than 10 years (50 (48.5%) trials in our study) are thus likely to be different in terms of surgical risk.

Figure 2, although appearing to show RCT cohorts lying within one standard deviation of our local reference population for our chosen demographic measures, also appears to show them lying systematically below the mean for BMI and weight. BMI has been shown to be linked to complications in surgical populations (Ri et al. 2018), but the direction of this relationship is unclear, with some evidence that obesity may be protective in abdominal surgery (Tjeertes et al. 2015). Regardless of direction, a change in population BMI, particularly when crossing thresholds for obesity (BMI > 30), seems to represent a change in patient risk, which supports our definition of substantial mismatch in BMI. If RCT participants fall into a different BMI category from those in the reference population (as seen in a quarter of trials in our study), they are thus likely to be different in terms of surgical risk, and applicability of trial results to our population is called into question.

The question of impact of the trials in this review is relevant. One potential weakness of our study is the inclusion of trials from over 30 years ago, which clinicians may dismiss out of hand as too old or irrelevant. However, each new RCT adds to work that came before, and eventually a meta-analysis is undertaken, hopefully demonstrating a significant treatment effect. Several highly cited perioperative meta-analyses include trials from 1988 to 1991 (9–11)—older studies are in this way impactful despite a high risk of mismatch to current patients. Newer trials can also be similar; current guidelines for enhanced recovery after colorectal surgery (ERAS) (Gustafsson et al. 2018) cite a 2018 trial (Leede et al. 2018) shown in our study to include patients significantly less overweight (lower BMI) and significantly less unwell (lower proportion of ASA 3 +) than those in our reference cohort. The assumption that newer trials include more representative patients appears to be true on average, but not always for every individual RCT.

We also included a majority of trials with recruitment conducted entirely outside the UK. This may be an explanation for mismatching of RCT cohorts to our UK-derived reference cohort. However, trials including UK centres are spread evenly across time in our set of included trials, with four published earlier than 13.4 years ago and five more recently. Moreover, evidence is now commonly used globally to inform treatment decisions—UK-based National Institute for Health and Care Excellence (NICE) guidelines on perioperative fluid management (Institute and for Health and Care Excellence. Perioperative care in adults: evidence review for intravenous fluid management strategy NICE guideline NG180 Perioperative care.^2020^. 2020) cite studies from the USA, Germany, China, and India. Time trends and differences in evidence will therefore be relevant regardless of the origin country of the evidence itself.

#### Implications and conclusions

Multicentre RCTs, and meta-analyses of them, are not-infrequently practice changing, and the conclusions are applied to current patients, who receive treatment accordingly. Our study suggests that many trials, particularly those published less recently, are not applicable to our local cohort of current patients and may therefore damage the applicability to this same cohort of a meta-analysis result. The same might be true of other local populations, if compared in the same way. Editorials have described an evidence ‘house of cards’ (Boyd 2016) and have noted a ‘reproducibility crisis’ (Baker and Penny 2016) across research domains, including anaesthesia and perioperative medicine (Gadsden 2023). The causes of a similar well-documented trend for reversal of study findings over time (Prasad et al. 2013) are certainly complex, but may in part be due to changes in population demographics, such that original trial populations no longer represent current populations, as we have shown.

Our results lead us to conclude that participants enrolled in trials of perioperative interventions in major abdominal surgery may be significantly different from those we currently treat in our centre. This effect appears most pronounced for ASA status and occurs more often in older trials—trials may thus ‘expire’ over time. The conclusions of these older trials, and meta-analyses and guidelines incorporating them, may not be relevant to our current patients undergoing major abdominal surgery, and associated treatment decisions should be weighed accordingly. Because these treatment decisions are made locally, in a clinician’s own hospital, reference cohorts should be developed locally and compared to trial evidence to inform on-the-ground clinical practice, rather than relying on comparisons to national registries (or assuming a good/bad match at face value). Our perioperative population (drawn from a London teaching hospital) appears poorly represented in RCTs, and the same may be true of populations in other hospitals. Checking the relationship of a multicentre or pooled trial cohort to an up-to-date local one may help interpretation and application of perioperative RCT results.

### Supplementary Information

**Additional file 1:** **Appendix 1.** Search Strategy.

## Data Availability

The datasets used and/or analysed during the current study are available from the corresponding author on reasonable request.
