# Supplementary material for: Mismatch of populations between randomised controlled trials of perioperative interventions in major abdominal surgery and current clinical practice
Source: Perioper Med (Lond). 2023 Nov 16;12:60. doi: 10.1186/s13741-023-00344-w (PMC10655289; doi:10.1186/s13741-023-00344-w)
Supplement: Supplementary file 1 — Additional file 1: Appendix 1. Search Strategy. [file 13741_2023_344_MOESM1_ESM.pdf]

Appendix 1

Search Strategy

Searches via Ovid, 28/09/2022

Strategy adapted from those used in:

1. Deng C, Bellomo R, Myles P. *Systematic review and meta-analysis of the perioperative use of vasoactive drugs on postoperative outcomes after major abdominal surgery.* British Journal of Anaesthesia, Volume 124, Issue 5, 2020, https://doi.org/10.1016/j.bja.2020.01.021.
2. Boet, S., Burns, J.K., Cheng-Boivin, O. *et al. Mapping multicenter randomized controlled trials in anesthesiology: a scoping review.* Syst Rev 10, 276 (2021). https://doi.org/10.1186/s13643-021-01776-5

Medline

1. colorectal surgery/ or general surgery/ or gynecology/ or urology/ or vascular surgery/ or aneurysm/
2. (surg* adj3 (colo* or general or gyn?eco* or urolog* or abdom* or liver or vascular or aneurysm)).tw.
3. 1 or 2
4. exp perioperative care/ or preoperative care/
5. (perioperative or intraoperative or preoperative or postoperative).mp.
6. 4 or 5
7. 3 and 6
8. randomized controlled trial.pt.
9. controlled clinical trial.pt.
10. randomized.ab.
11. placebo.ab.
12. clinical trials as topic.sh.
13. randomly.ab.
14. trial.ti.
15. 8 or 9 or 10 or 11 or 12 or 13 or 14
16. exp animals/ not humans.sh.
17. 15 not 16
18. limit 7 to "all adult (19 plus years)"
19. 17 and 18
20. Multicenter Studies as Topic/
21. multicenter study.pt.
22. multicenter study/
23. multi?cent*.tw.
24. 20 or 21 or 22 or 23
25. 19 and 24

Embase

1. exp aortic surgery/
2. exp pelvis surgery/
3. exp abdominal surgery/
4. exp urologic surgery/
5. general surgery/
6. (surg* adj3 (aort* or aneurysm or pelvi* or abdom* or general or gyn?eco* or urolog* or liver)).tw.
7. 1 or 2 or 3 or 4 or 5 or 6
8. perioperative period/
9. exp intraoperative period/
10. preoperative period/ or exp premedication/ or preoperative care/ or preoperative treatment/
11. postoperative period/ or mobilization/ or postanesthesia care/ or postoperative care/ or exp wound healing/
12. ((period or phase) adj3 (periop* or perop* or intraop* or preop* or postop*)).mp. [mp=title, abstract, heading word, drug trade name, original title, device manufacturer, drug manufacturer, device trade name, keyword heading word, floating subheading word, candidate term word]
13. 8 or 9 or 10 or 11 or 12
14. 7 and 13
15. (random$ or factorial$ or crossover$ or cross over$ or cross-over$ or placebo$ or (doubl$ adj blind$) or (singl$ adj blind$) or assign$ or allocat$ or volunteer$).mp. [mp=title, abstract, heading word, drug trade name, original title, device manufacturer, drug manufacturer, device trade name, keyword heading word, floating subheading word, candidate term word]
16. crossover procedure/ or double blind procedure/ or randomized controlled trial/ or single blind procedure/
17. 15 or 16
18. 14 and 17
19. limit 18 to (adult <18 to 64 years> or aged <65+ years>)
20. limit 14 to ((randomized controlled trial or controlled clinical trial) and (adult <18 to 64 years> or aged <65+ years>))
21. 19 or 20
22. "multicenter study (topic)"/
23. multicenter study/
24. 22 or 23
25. 21 and 24
